# Voice and Swallowing Outcomes Following Airway Reconstruction in Adults: A Systematic Review

**DOI:** 10.1002/lary.28494

**Published:** 2020-01-13

**Authors:** Gemma M. Clunie, Justin W. G. Roe, Caroline Alexander, Gurpreet Sandhu, Alison McGregor

**Keywords:** Laryngotracheal stenosis, laryngotracheal reconstruction, voice, swallowing

## Abstract

**Objectives:**

Laryngotracheal stenosis is a rare condition characterized by upper airway narrowing. Reconstructive surgical treatment aims to manage the area of stenosis to improve dyspnea and can impact voice and swallowing function. This article critically evaluates the literature about voice and swallowing outcomes in adults with laryngotracheal stenosis who undergo reconstructive surgery.

**Study Design:**

Systematic review.

**Methods:**

Six databases were searched for articles referring to voice and swallowing outcome measures following reconstruction procedures in adults with laryngotracheal stenosis. Screening was completed using predefined inclusion/exclusion criteria.

**Results:**

A total of 143 abstracts were reviewed, with 67 articles selected for full‐text review. Twenty studies met the inclusion criteria. Data extraction was completed with the Strengthening Reporting of Observational Studies in Epidemiology checklist with Oxford Centre for Evidence‐Based Medicine Level of Evidence used to indicate quality. Risk of bias was assessed using the Risk of Bias Assessment Tool for Non‐Randomized Studies. All studies scored a high risk of bias in at least one of the domains. Selection and timing of outcome measures was heterogenous, and there was limited information provided about rationale or reliability.

**Conclusions:**

The literature acknowledges the importance of voice and swallowing outcomes following airway reconstruction. Studies show correlation between reconstructive surgery and deterioration in vocal function; there are no consistent data about swallowing outcomes. The lack of a core outcome measures set for adults with laryngotracheal stenosis limits the findings of this review. Further research is needed to establish clear criteria for robust and clinically relevant outcome measurement. *Laryngoscope*, 131:146–157, 2021

## INTRODUCTION

Laryngotracheal stenosis is a rare condition characterized by a narrowing of the airway at any point between the supraglottis and the carina.^1^ In adults, 80% of cases are acquired, with the most common cause being postintubation injuries and prolonged ventilation on intensive care units (an estimated incidence of one in 200,000).^2^ Other etiologies include postradiotherapy changes, malignancies and autoimmune conditions, for example sarcoidosis or granulomatosis with polyangiitis.^3^ Another subgroup is patients who suffer from idiopathic subglottic stenosis, a progressive condition often misdiagnosed as asthma. This predominantly affects Caucasian women between 40 and 50 years old and results from a gradual fibroinflammatory process.^4^

Despite the multifactorial nature of the condition, the presenting symptoms are consistent amongst patient groups and include breathlessness, stridor, as well as voice and swallowing difficulties.^1^, ^5^ Patients frequently require tracheotomies due to the reduced patency of their airway and may also be treated with repeated endoscopic procedures to manage the stenosis. If this no longer helps, they require more complex and innovative surgeries, for example cricotracheal resection (CTR) or laryngotracheal reconstruction (LTR).^6^

For adults, research in this area has focused on the primary surgical outcomes of improved airway patency and breathing difficulties, with changes to voice and swallowing typically considered as secondary outcomes.^7^ The involvement of the fragile structures of the supraglottis, larynx, and subglottis, combined with the complexity of the surgical reconstruction, means that voice and swallowing difficulties are frequently observed^7^, ^8^, ^9^ even when dyspnea has improved,^10^, ^11^ but the details of if, how, and when voice and swallowing are affected by reconstructive surgery remain unclear. To date, there has been no review of this literature in adults.

Recent articles have begun to ask more questions about these functional outcomes of reconstructive airway surgery^10^, ^11^, ^12^ and acknowledge that although the key aim for patients and clinicians is to improve their breathing, other changes to their day‐to‐day function need to be taken into account both in preoperative counseling and postoperative follow‐up.^11^ This is particularly pertinent for speech–language pathologists working with this population who must be able to base current practice and advice on the best available evidence.

This systematic review uses a population, intervention, comparison, outcome framework^13^ to identify studies of adults with laryngotracheal stenosis (population) who have undergone reconstructive surgery (intervention) where changes to voice and swallowing (outcome) have been considered. The review is specifically designed to clarify the following: 1) determine the quality and relevance of the research completed to date; 2) the detail available to clinicians/speech–language pathologists about changes to voice and swallowing because of reconstructive surgery; 3) identify gaps in the literature; and 4) help guide the direction of future research.^14^

## METHODS

The Preferred Reporting Items for Systematic Reviews and Meta‐analysis (PRISMA) guidelines were used to carry out the systematic review.^15^ The protocol was registered on PROSPERO, an International prospective register of systematic reviews, on October 25, 2018 (CRD42018108316).

### Search Strategy

#### Identification of studies

Key search terms were classed as “airway stenosis,” “laryngotracheal stenosis,” “subglottic stenosis,” and “tracheal stenosis.” Alternative terms were identified using Medical Subject Headings, through peer discussion, and checking keyword lists of relevant published studies. The strategy was tested and refined in Embase. The list of search terms used is listed in Table 1. An electronic search of databases was completed of the Allied and Complementary Medicine Database, Cumulative Index to Nursing and Allied Health Literature, Embase, and MEDLINE between July 31, 2018 and August 8, 2018. The search was completed by the lead author and repeated for completeness on June 12, 2019. No limits were placed in relation to publication status, years since publication, or language.

**TABLE 1:** Search Terms.

Category 1	Category 2	Category 3 (OR)	Category 4
Subglottic stenos?s	Laryngotracheal reconstruction	Swallow*	Adult
Tracheal stenos?s	Laryngotracheal resection	Dysphagia	
Airway stenos?s	Cricotracheal reconstruction	Deglutition	
Laryngotracheal stenos?s	Cricotracheal resection	Voice	
	Tracheal reconstruction	Dysphonia	
	Tracheal resection, airway reconstruction, airway resection	Hoarse*	
	Maddern procedure		

? and * correspond to search wildcards to include all variations.

Grey literature was also reviewed using a Google internet search and a search of OpenGrey and National Health Service (NHS) Digital databases.

#### Article screening

**Fig 1:**
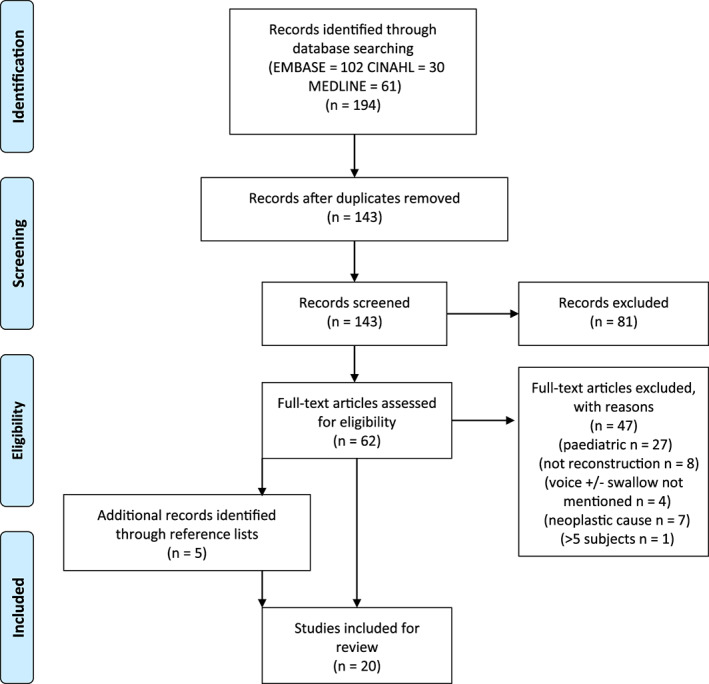
Preferred Reporting Items for Systematic Reviews and Meta‐analyses (PRISMA) flowchart showing the screening and eligibility process using inclusion/exclusion criteria. CINAHL = Cumulative Index to Nursing and Allied Health Literature. [Color figure can be viewed in the online issue, which is available at www.laryngoscope.com.]

The initial database searches retrieved 194 results, which were collected into a reference manager (EndNote). Following removal of duplicates (51) the articles from the search were assessed for their inclusion and exclusion criteria by the first author (g.c.). The inclusion criteria were as follows: 1) study involved human participants ≥18 years of age, 2) English‐language articles only, 3) laryngotracheal stenosis diagnosis confirmed by any diagnostic criteria, 4) reconstructive surgery involved, 5) swallowing and/or voice referred to in the article, 6) does not involve patients with active malignancy, and 7) case series involves n ≥ 5. These criteria were applied to the titles and abstracts of the electronic search, and articles that did not meet them were excluded. Articles were included for full‐text review where it was not possible to use the abstract to fully assess their eligibility. The full text of 62 articles were then retrieved and assessed using the inclusion and exclusion criteria to ensure that the relevant studies were included in the review. Any ambiguities or discrepancies were resolved by discussion with three of the other authors (c.a., j.w.g.r., g.s.). The reference lists of the full‐text articles were then screened by the first author (g.m.c.) for completeness (this resulted in five extra articles). The PRISMA flow diagram^15^ used for this systematic review is shown in Figure 1. Once this process was completed, 20 titles were selected for extraction and analysis.

#### Extraction, quality assessment, and risk of bias

Data extraction was completed by one reviewer (g.m.c.) using The Strengthening Reporting of Observational Studies in Epidemiology (STROBE)^16^ checklist. This was chosen due to its use as a guide for reporting observational studies, because most of the articles reviewed were within this category.^17^ Main details are summarized in Table 2 and include study design and the Oxford Centre for Evidence Based Medicine (OCEBM) level of evidence,^18^ number of participants, age, gender, type of stenosis, type of reconstruction, and the voice and/or swallowing outcome measure. Due to the heterogeneity of the outcome measures used, it was not possible to provide summary statistics or responsiveness to change for all except four of the studies. Therefore, other key results have been descriptively summarized and reviewed (Tables 3 and 4) with consideration of selection rationale, validity, and reliability where appropriate.

**TABLE 2:** Summary of Included Studies.

Authors	Year	No.	Study Type	OCEBM Level of Evidence	Cause of Stenosis	Type of Surgery	Patient Group	Outcome Measured: Voice/Swallow/Both	Primary/Secondary Outcome Measure
Bryans et al.^8^	2013	23	Cohort	IV	Mixed	CTR	Women 37–86 years	Voice	Primary
Daneshi et al.^53^	2010	10	Case series	IV	Intubation	Two‐stage LTR	Men 18–30 years	Voice	Secondary
Grillo et al.^28^	1992	49	Case series	IV	iSGS	LTR	Men and women 18–70+ years	Voice	Secondary
Hashemi et al.^57^	2016	52	Case series	IV	Intubation	TR	Men and women 20–80+ years	Voice	Secondary
Houlton et al.^27^	2011	16	Case series	IV	Mixed	CTR	Men and women 29–67 years	Voice	Primary
Marulli et al.^54^	2008	37	Case series	IV	Intubation and iSGS	LTR/CTR	Men and women 18–71 years	Voice	Secondary
Menapace et al.^42^	2017	33	Case series	IV	iSGS	LTR/CTR/TR	Men and women 31–71 years	Voice	Secondary
Smith et al.^22^	2008	14	Case series	IV	iSGS	CTR	Women 35–69 years	Voice	Primary
Tanner et al.^23^	2017	11	Case series	IV	iSGS	Revised CTR	Women 33–73 years	Voice	Primary
Terra et al.^30^	2009	20	Case series	IV	Intubation	LTR or CTR	Men and women 18–54 years	Voice	Secondary
Casiano et al.^34^	1994	9	Case series	IV	Intubation	LTR	Men and women 18–71 years	Swallow	Secondary
Kim et al.^55^	2017	36	Case series	IV	Intubation	LTR or TR	Men and women No age range given 47 years (mean)	Swallow	Secondary
Lennon et al.^9^	2016	38	Case series	IV	Mixed	LTR	Men and women 20–80 years	Swallow	Primary
Merati et al.^56^	2005	17	Case series	IV	Intubation and iSGS	TR	Men and women 23–76 years	Swallow	Secondary
Fiz et al.^11^	2018	44	Case series	IV	iSGS	CTR	Women 31–79 years	Both	Primary
Liberman et al.^35^	2009	18	Case series	IV	Intubation and iSGS	LTR	Men and women 20–75 years	Both	Secondary
Morcillo et al.^29^	2013	64	Case series	IV	iSGS	LTR	Men and women 19–77 years	Both	Secondary
Rich et al.^36^	2016	11	Case series	IV	Mixed	LTR	Men and women 22–71 years	Both	Secondary
Sittel et al.^21^	2008	15	Case series	IV	Mixed	CTR	Men and women 24–76 years	Both	Secondary
van den Boogert et al.^31^	2004	10	Case series	IV	Mixed	LTR or CTR	Men and women 19–61 years	Both	Secondary

CTR = cricotracheal resection; iSGS = idiopathic subglottic stenosis; LTR = laryngotracheal reconstruction; OCEBM = Oxford Centre of Evidence Based Medicine; TR = tracheal resection.

**TABLE 3:** Voice Outcomes: Type, Timing, Reliability, and Key Findings.

	Type of Outcome Measure						Rationale	Timing of Outcome Measurement	Reliability	Key Details
	Auditory‐perceptual	Visuo‐perceptual	Acoustic	Aerodynamic	Patient‐Reported	Other				
Bryans et al.^8^	X	X	X		X		NS	Postsurgery 33.2 months (SD = 19.4 months; range, 11–70 months).	Auditory‐perceptual measures: Intrajudge agreement (*r*) score = 0.92 (range, 0.78–0.99 across six domains), patient‐reported subjective scales *r* = 0.78–0.87	Postsurgery (between subject–CTR compared to dilatation group): acoustic measures: mean F0 Hz 188.13 ± 14.42, *P* = .01; max F0 Hz = 298.38 ± 48.06, *P* < .01; pitch range = 13.33 ± 2.56, *P* < .01; minimum loudness Hz = 56.00 ± 4.58, *P* = .02; maximum loudness Hz = 86.78 ± 5.81, *P* < .01. CAPE‐V (clinician rated): pitch = 8.78 ± 13.08, *P* = .05; loudness = 13.67 ± 20.79, *P* = .01; breathiness = 15.89 ± 22.08, *P* = .06. patient reported: VHI total = 39.69 ± 30.66, *P* < .01; no. of voice symptoms = 6.18 ± 4.14, *P* = .01; voice‐related satisfaction = 6.09 ± 2.84, *P* = .02
Daneshi et al.^53^						X	NS	Postsurgery 1 year	NS	100% of patients had satisfactory voice (glottic stenosis excluded)
Grillo et al.^28^						X	NS	Postsurgery (1–15 years)	NS	Postsurgery: 20% excellent (n = 10), 44% good (n = 22), 4% fair (n = 4), 2% failure (n = 1)
Hashemi et al.^57^						X	NS	Postsurgery not reported	NS	Postsurgery: 5.8% hoarseness (n = 3/52), 15.4% vocal cord dysfunction (n = 8/52)
Houlton et al.^27^	X		X	X			NS	Presurgery (10/16), postsurgery (13/16) follow‐up range 4.3 months (range, 0.7–13.2 months)	Auditory perceptual measures: ICC 0.55–0.94 (across six domains)	Comparison pre‐ and postsurgery (within subject): mean F0 Hz: preoperative = 206.5 ± 13.8, postoperative = 151.1 ± 32.3, *P* = .002; mean F0 connected speech: Preoperative = 194.9 ± 23.1, postoperative = 152.7 ± 27.4, *P* = .047
Marulli et al.^54^						X	NS	Postsurgery median = 6.5 years (range, 7 months–13 years)	NS	Postsurgery: 2.7% severe hoarseness and reduction in volume (n = 1/37); also described “deepening in tone of voice” in discussion
Menapace et al.^42^						X	NS	Postsurgery average = 9.7 years	NS	Postsurgery: 21% excellent (n = 7/33), 47% good (n = 15/33), 16% fair (n = 5/33), 16% poor (n = 5/33), 3% no information (n = 1/33)
Smith et al.^22^			X		X		NS	Presurgery and postsurgery voice recording 3.5 months (range, 2.4–5.2 months), postsurgery VHI 11 months (range, 3–32 months	NS	Comparison pre‐ and postsurgery: Acoustic, mean spoken F0, preoperative = 186 Hz, postoperative = 165 Hz, *P* = .04; mean sustained vowel frequency, preoperative = 216 Hz, postoperative184 Hz, *P* = .03; F0 range reduced 21.5–15.6, *P* = .05; highest frequency from pitch glide, preoperative = 537 Hz, postoperative = 391 Hz, *P* < .05
Tanner et al.^23^	X		X		X		NS	Presurgery and postsurgery	Auditory‐perceptual measures: ICC single measures = 0.70, ICC average measures = 0.963, *P* > .01 between raters	Comparison pre‐ and postsurgery: acoustic, mean spoken F0, preoperative = 215 Hz ± 40 Hz, postoperative = 201 Hz ± 65 Hz, *P* = .376 not statistically significant (paired *t* test Bonferroni correction 0.05/9, *P* = .005); auditory perceptual, judgment of voice disorder severity (sustained vowel), presurgery = 36.5/100 ± 23.2, postsurgery 33.5/100 ± 29.4, *P* = .621 not statistically significant; patient‐reported VHI scores decreased–unable to compare due to missing data at pretreatment stage
Terra et al.^30^						X	NS	Postsurgery mean = 28 months (range, 27–29 months)	NS	Postsurgery: assessment, 25% dysphonia characterized by low pitch, hoarseness and reduced vocal intensity (n = 5/20), laryngoscopy (of the 25%), described anatomic alterations, vocal fold asymmetry, misapproximation during closure
Fiz et al.^11^						X	NS	Presurgery; postsurgery variable follow‐up	NS	Pre‐ and postsurgery comparison: preoperative = 1.34 ± 0.71, postoperative = 2.91 ± 0.42, *P* < .001; “Majority of patients passed from slight or absent voice problems to post‐operative dysphonia due to cricothyroid muscles and cricoid arch resection with ensuing loss of vocal pitch modulation. As a result, most subjects experienced significant difficulties in being heard or understood in loud environments.”
Liberman et al.^15^					X		NS	Presurgery 18/18, Postsurgery 17/18, 9.1 ± 1.2 months (range, 2–17 months)	NS	11.8% dysphonia (n = 2/18)
Morcillo et al.^29^							NS	Postsurgery at least 1 year	NS	Postsurgery: 17% (n = 10/60) dysphonia
Rich et al.^36^					X		NS	Postsurgery mean = 76 months; median = 75 months (range, 18–162 months)	NS	Postsurgery: VHI‐30 mean score = 34, median score = 32 (range 21–58), consistent with mild dysphonia
Sittel et al.^21^							NS	Postsurgery late sequela	NS	Postsurgery: 57% (n = 8/15) lower pitch
van den Boogert et al.^31^						X	NS	Post surgery mean = 15 months (range, 8–53 days)	NS	77% (n = 10/14) good, 23% (n = 3/14) satisfactory

Acoustic = phonation time, fundamental frequency, pitch range, vocal loudness; Aerodynamic = perturbation measures, subglottic and glottic airflow; Auditory‐perceptual = Consensus Auditory Perceptual Evaluation of Voice (CAPE‐V); CTR = cricotracheal resection; F0 = fundamental frequency; ICC = intraclass correlation coefficient; NS = not stated; Other = otolaryngologist report; satisfaction scale; laryngoscopy; Airway‐Dyspnoea‐Voice‐Swallowing Scale; Patient reported = Voice Handicap Index (VHI); SD = standard deviation; Visuo‐perceptual = clinician analysis of phonation tasks during endoscopic evaluation of voice.

**TABLE 4:** Swallowing Outcomes: Types, Timing, Reliability and Key Findings.

	Type of Outcome Measure				Rationale	Timing of Outcome Measurement	Reliability	Key Details
	Surrogate Measures	Instrumental	Patient‐reported	Other				
Casiano et al.^34^				X	NS	Postsurgery 6–30 months	NS	Postsurgery: 0% (n = 0/9) had dysphagia
Kim et al.^19^		X	X		NS	Postsurgery 2–6 months	NS	Postsurgery: 2.6% complained of food getting stuck (n = 2/36), both MBS = normal but patients continued to complain of difficulties for several months postsurgery
Lennon et al.^9^	X	X			NS	Postsurgery up to 6 months	NS	Postsurgery: no. of days of dysphagia symptoms (mean and SD), all patients = 8 ± 27.2 days, no stent placed = 4.8 ± 5.3 days, with stent following stent removal = 11.1 ± 40.7 days, with stent + feeding tube = 50.8 ± 53.6 days, with stent + no feeding tube 3.7 ± days; comparison between: without grafts = 10.8 ± 33.1 days, *P* = .35; videofluoroscopy: 94% patients with stent had VF (n = 16/17), 37.5% moderate or moderate–severe, dysphagia + aspiration (n = 6/17), 31% mild dysphagia/silent aspiration (n = 5/17), 31% trace penetration only (n = 5/17), with grafts = 2.2 ± 5.7 days
Merati et al.^56^	X				NS	Postsurgery 2 months–6 years	NS	Postsurgery: 88% (n = 14/17) started oral feeding day 1 postoperatively
Fiz et al.^11^				X	NS	Presurgery; postsurgery variable follow‐up	NS	Pre‐ and postsurgery comparison: preoperative 1.11 ± 0.32, postoperative 1.57 ± 0.73, *P* < .001, “Majority of patients showed onset of mild subjective swallowing difficulties even thought they were able to eat a normal diet”
Liberman et al.^35^			X		NS	Presurgery 18/18; postsurgery 17/18, 9.1 ± 1.2 months (range, 2–17 months)	NS	Preoperative dysphagia = 1.8 ± 3.3, postoperative dysphagia = 1.1 ± 2.4, *P* = .226
Morcillo et al.^29^					NS	Postsurgery at least 1 year	NS	Postsurgery: 5% (n = 3/60) aspiration, 1.7% (n = 1/60) dysphagia
Rich et al.^36^			X		NS	Postsurgery, mean = 76 months; median = 75 months (range, 18–162 months)	NS	Postsurgery: MDADI mean score = 93.7, median score = 92.5 (range, 89–100), consistent with no dysphagia
Sittel et al.^21^					NS	Postsurgery late sequela	NS	Postsurgery: 100% (n = 15/15) dysphagia
Van den Boogert et al.^31^				X	NS	Postsurgery mean = 15 months (range 8–53 days)	NS	8% (n = 1/14) dysphagia

Instrumental = videofluoroscopy or modified barium swallow; NS = not stated; Other = otolaryngologist report, satisfaction scale, laryngoscopy, Airway‐Dyspnoea‐Voice‐Swallowing Scale; Patient reported = M. D. Anderson Dysphagia Inventory (MDADI); SD = standard deviation; Surrogate measures = duration of dysphagia symptoms; commencement of oral feeding postsurgery.

The Risk of Bias Test for Non‐Randomized Studies (RoBANS)^19^ was chosen for assessment of study design and risk of bias (Table 5). This tool was specifically designed for the assessment of nonrandomized studies within systematic reviews, including case series, and was selected due to its compatibility with the Cochrane standard and potential for broad usage.^19^, ^20^ Using the RoBANS allows the studies to be assigned a low, high, or unclear risk of bias for six domains: participant selection, confounding variables, measurement of exposure, blinding of outcome assessment, incomplete outcome data, and selective outcome reporting. The focus of this systematic review was voice and swallowing outcomes; therefore, to be consistent with the aims of the review, risk of bias assessment was applied to these outcomes, and not dyspnea.

**TABLE 5:** Summary of Risk of Bias Assessment Using RoBANS Assessment.

Study	Participant Selection (Selection Bias Caused by the Inadequate Selection of Participants)	Confounding Variables (Selection Bias Caused by the Inadequate Confirmation and Consideration of Confounding Variable)	Measurement of Exposure (Performance Bias Caused by the Inadequate Measurement of Exposure)	Blinding of Outcome Assessment (Detection Bias Caused by the Inadequate Blinding of Outcome Assessments)	Incomplete Outcome Data (Attrition Bias Caused by the Inadequate Handling of Incomplete Outcome Data)	Selective Outcome Reporting (Reporting Bias Caused by the Selective Reporting of Outcomes)	Overall Risk of Bias
Bryans et al.^8^	High	Low	Low	Low	Low	Low	Low
Casiano et al.^34^	High	High	Low	High	Unclear	High	High
Daneshi et al.^53^	High	High	Unclear	High	Low	High	High
Fiz et al.^11^	High	High	Low	Low	Low	Low	High
Grillo et al.^28^	High	High	Low	High	Low	High	High
Hashemi et al.^57^	High	High	Low	High	Low	High	High
Houlton et al.^27^	High	Low	Low	Low	High	Low	High
Kim et al.^19^	High	High	Low	High	Low	High	High
Lennon et al.^9^	High	High	Low	Unclear	Low	Low	High
Liberman et al.^35^	High	High	Low	Unclear	Low	Low	High
Marulli et al.^54^	High	High	Low	High	Low	High	High
Menapace et al.^42^	High	High	Low	High	High	High	High
Merati et al.^56^	High	High	Low	High	Low	High	High
Morcillo et al.^29^	High	High	Low	High	Low	Low	High
Rich et al.^36^	High	High	Low	Low	High	Low	High
Sittel et al.^21^	High	Unclear	Low	Low	High	Low	High
Smith et al.^22^	High	Low	Low	Low	Low	Low	Low
Tanner et al.^23^	High	Low	Low	Low	Low	Low	Low
Terra et al.^30^	High	High	Low	High	Low	High	High
van den Boogert et al.^31^	High	High	Low	High	Low	Low	High

Because the purpose of this systematic review was to focus on the voice and swallowing outcome measurement of the retrieved articles, risk of bias assessment has focused on these outcomes, whether primary or secondary, and not outcomes relating to dyspnea.

RoBANS = Risk of Bias Assessment Tool for Non‐Randomized Studies.

It was not appropriate to apply a quality threshold to inclusion for the purposes of this review because the retrieved articles were all observational, and the aim of the review was not to make recommendations about care, but to provide an overview of the current literature.

A second reviewer (j.r.) reviewed 50% of the articles (every other) in the same manner. Any discrepancy was adjudicated by another author (c.a.).

## RESULTS

### Critical Appraisal of Studies

The OCEBM levels of evidence^18^ for each study are presented in Table 2. These show that of the 20 final articles, 19 present data from case series—evidence level 4. One article is a cohort design, but due to the retrospective design, is also downgraded to level 4.

A summary of the risk of bias assessment is given in Table 5. According to RoBANS guidelines, each study was assessed as high or low risk of bias for each of the six domains, with a rating of unclear used when this was uncertain due to lack of information within the text. An overall risk of bias was determined by reviewing the classification of three key domains: participant selection, confounding variables and incomplete outcome data. Overall risk of bias was judged as high, low or unclear if “more than one of the three key domains was assessed as having a low, unclear, or high risk of bias.”^19^

All studies received a high risk of bias for participant selection. For 19 of the articles this was because they were retrospective case series. One study did have a cohort design with comparison of voice outcomes made between patients who had undergone CTR versus dilatation. However, the study was retrospective, and there was no randomization or matching possible between the two groups.^8^

The single prospective study^21^ was a case series and did not report a sample size calculation. Recruitment was based on their inclusion criteria, the timeframe, and records available. This can lead to risk of bias within the sampling population.

For the other five categories of bias there was more variability, with three studies scoring low risk of bias across the remaining domains^8^, ^22^, ^23^ due to their use of objective voice outcome measures, with no missing data and appropriate blinding of voice assessment parameters. These three studies were classified as a low overall risk of bias. Eleven of the studies were classified as low risk of bias for selective outcome reporting; however, due to the likelihood of confounding variables (in the case of surgical case series an expectation of learning effect across the course of data collection), they could not be rated as low risk of bias overall.

### Analysis of Outcome Measures

To allow for analysis of the literature, voice outcome measures were grouped into the five main categories commonly used.^24^, ^25^ Similarly, swallowing outcome measures have been grouped into three categories in keeping with the literature.^26^ Other has been added as an extra category to both groups to account for descriptive measures.

Tables 3 and 4 show a breakdown of the type of voice and swallowing outcome measure used, including the time points of assessment and any information provided on selection rationale or reliability of the measure. Although the STROBE checklist asks for the reporting of the validity of outcome measures, this was not included in the analysis because none of the outcome measures used have been validated on a population of adults with laryngotracheal stenosis.

A total of 80% of the studies reported details on voice outcome measures. Of these, four studies (20%) analyzed voice outcomes as their primary measure. Fewer studies (50%) reported on swallow outcomes within their analysis, with only one (5%) using swallowing outcomes as their primary measure. Six studies (30%) reported data on both swallow and voice outcomes, and one article (5%) analyzed voice and swallowing as its primary outcome measure (concomitantly with airway and dyspnea assessment). The remaining 70% considered voice and swallowing outcomes as a secondary measure, with surgical outcomes the primary concern.

Only four of the studies^11^, ^22^, ^23^, ^27^ included a presurgical time point of assessment to allow for within category comparison of outcome, and this was not always achieved for every participant. Postsurgical time points of assessment were given in 80% of the articles but varied greatly between studies (range, 0.7 months to 15 years). None of the studies provided a rationale for the voice and swallowing outcomes chosen.

Three studies^8^, ^23^, ^27^ provided reliability data specifically for a voice outcome, the auditory‐perceptual outcome measurement, with one also reviewing the reliability of the patient‐reported voice symptoms subjective scale. This did not relate to the reliability of the tool for use with the population, but for the intrarater reliability of the clinicians, and therefore has limited clinical applicability.

### Detail of Outcome Measures and Descriptive Analysis

Details of the different patient groups, outcome measures and key results given in each of the articles have been presented in Table 3 for voice outcomes, and Table 4 for swallowing outcomes. This varies from descriptive text to statistical analysis due to the heterogeneity of the outcome measures used in the studies.

### Voice

The main category of voice outcome measure was Other (40%), representing either a satisfaction scale (15%) as described by Grillo et al. in their early work on laryngotracheal stenosis^28^ or clinician report of perceptual voice quality (25%). This was consistent with these articles referring to voice as a secondary outcome and not looking for detailed information. However, 100% of the articles that considered voice as an outcome reported deterioration in voice quality postsurgery, and four articles referenced patients receiving voice therapy for their difficulties.^21^, ^29^, ^30^, ^31^ Fiz et al. used the Airway‐Dyspnoea‐Voice‐Swallow (ADVS) scale to demonstrate a statistical reduction in voice quality postsurgery (see Table 3 for detail).

Table 6 shows a comparison between the four studies that specifically reviewed voice changes pre‐ and post‐CTR surgery. This updates a similar table created by Bryans et al.^8^ The methodology and patient population for each study varied (although the cohort was 98% female [63/64]), with different outcome measures used despite similar aims. However, reduction in fundamental frequency, reduction in patient‐related quality‐of‐life scores, and reduced pitch range seem to be consistently observed within the three articles^8^, ^22^, ^27^ that reviewed outcomes for patients following standard CTR surgery, where infrahyoid release takes place and the cricothyroid membrane is transected.^32^, ^33^ Tanner et al. explored the voice outcome measures for patients following a revised voice‐sparing CTR procedure where the cricothyroid membrane is left intact. This procedure seems to demonstrate less impact on fundamental frequency and improved patient‐reported scores.^23^

**TABLE 6:** Comparison of Studies Focusing on Voice Outcomes after CTR.

	Smith et al.		Houlton et al.		Bryans et al.		Tanner et al.	
	Pre‐CTR	Post‐CTR	Pre‐CTR	Post‐CTR	Dilation	Post‐CTR	Pre‐Revised CTR	Post‐Revised CTR
Maximum phonation time (sec)	7.7	8.7	11.7	12.5	16.35	14.40		
Mean fundamental frequency /α/ (Hz)	216	184	203.2	157.1	214.05	188.13	214.9	201.0
Mean fundamental frequency speech (Hz)	186	165	195	157.9	185.64	172.45		
Pitch range /α/ (semitones)	21.5	15.6	24.2	18.6	23.30	13.33		
CAPE‐V overall			27.8	47.5	6.67	19.78		
VHI‐30 total score	NR	21.9			6.29	39.69	34.0	17.2

CAPE‐V = Consensus Auditory‐Perceptual Evaluation of Voice; CTR = cricotracheal resection; NR = not reported; VHI‐30 = Vocal Handicap Index 30.

### Swallowing

The data available on swallowing outcomes following reconstructive surgery were minimal, with little consistency between articles in terms of what was measured and how it was measured. The most commonly used outcome measures were other and patient‐reported (15%, respectively), with instrumental assessment and surrogate measures referred to in 10% of the studies.

There was significant variation between articles in terms of swallowing outcome results. Three articles reported no significant swallowing difficulties in their patients.^34^, ^35^, ^36^ The remaining articles acknowledged the potential for swallowing to be affected by reconstructive surgery for laryngotracheal stenosis; however, minimal detail is provided as to the nature, severity, or duration of swallowing difficulties.

The two studies (10%) that used swallowing as a primary outcome^9^, ^11^ used two different outcome measures. Lennon et al.^9^ used duration of dysphagia symptoms postreconstruction. This was considered for the whole case series and compared according to the absence of a stent and graft as part of the LTR. Statistical analysis was descriptive, providing the mean duration of dysphagia symptoms in patients without stents and following stent removal (8 days; standard deviation [SD] = 27.2 days; median = 1.5 days) and comparing to patients without stents (4.8 days; SD = 5.3 days; median = 4 days). This showed that patients without stents had “shorter duration of dysphagia symptoms than those with stents.” The statistical analysis was also limited by the presence of an outlier (a patient who had a postoperative cerebrovascular accident). Ninety‐four percent of patients who had a stent were given an instrumental assessment (videofluoroscopy) as the measurement of swallowing difficulties (moderate or moderate to severe dysphagia) and was used to help clinical decision making, for example compensatory strategies and need for feeding tube.

Fiz et al.^11^ used the ADVS scale. This study demonstrated a significant decrease in swallowing score postoperatively (Table 4), although it states that patients were able to eat and drink normally. Conversely, two articles used patient‐reported questionnaires as secondary outcome measures and reported no significant change to swallowing because of the surgery.^35^, ^36^

## DISCUSSION

This systematic review evaluated 20 articles that referenced voice and swallowing outcomes following airway reconstruction for adults with laryngotracheal stenosis. The studies were reviewed 1) to determine the quality and relevance of the research completed to date, 2) the detail available to clinicians/speech–language pathologists about changes to voice and swallowing because of reconstructive surgery, 3) identify gaps in the literature, and 4) help guide the direction of further research.

The main findings of this review are that although voice and swallowing are being considered as relevant primary and secondary outcomes to patients with laryngotracheal stenosis who have reconstructive surgery, there is no consistent approach to the selection or timing of the outcome measures selected. The overall quality of the evidence derived from the 20 studies is very low and cannot be used to determine how voice and swallowing are affected by reconstructive surgery. This is due to the retrospective design of the studies limiting the information available to clinicians. The conclusions that can be drawn from the data when applying it to a clinical population are limited.

Seventy percent of the studies that met our selection criteria only considered voice and swallowing as secondary outcomes. This is consistent with the primary, and understandable, goal of reconstructive surgery for laryngotracheal stenosis being to improve patient's respiratory function and to achieve decannulation.^37^, ^38^ However, the variability and inconsistency of how these secondary outcomes have been reported limits their relevance to clinicians. It also does not acknowledge the importance of all functional outcomes to patients when making treatment decisions. In a recent pilot study, postoperative voice quality was identified as a highly significant factor to patients in determining whether to consider open reconstructive procedures.^39^

The remaining 30% of the studies where voice and/or swallowing outcomes were the primary focus of the study have similar variability between outcome selection and relevance. There are data regarding repeatability within the literature of certain voice outcome measures (acoustic and aerodynamic measures, auditory perceptual analysis, and patient‐reported measures^8^, ^22^, ^23^, ^27^). However, the outcome measures chosen in these studies lack validity and data around reliability. There are no validated outcome measure for this population, although the ADVS is a scale designed specifically for evaluation of patients with laryngotracheal stenosis.^40^ It is not consistently used across surgical centers and has only been validated on the pediatric laryngotracheal stenosis population.^41^

However, the body of evidence available for voice outcomes does demonstrate clinically useful information for patients and clinicians in relation to CTR and revised CTR procedures. Key points include reduction of pitch and loudness postsurgery, with a reduction in patient‐related satisfaction in their voices. Whether this is generalizable to other surgical techniques is not indicated by the literature; however, there was correlation within the literature that reconstructive surgery does lead to deterioration in voice function for some patients despite an improvement in dyspnea scores.^11^, ^42^

In terms of swallowing outcomes, there is no consistent evidence about the impact of reconstructive surgery on swallowing. The studies where swallowing is considered as a secondary measure offer contradictory, binary findings (swallowing difficulties or no swallowing difficulties). The two studies that consider swallowing as a primary outcome measure both show that swallowing is affected following surgery, but neither offer necessary detail or validity for clinical decision making or patient counseling.

For example, it can be hypothesized that swallowing could be more adversely affected by an LTR procedure compared to CTR. This is due to the necessary disruption an LTR causes to the laryngeal framework as acknowledged in pediatric literature.^43^, ^44^, ^45^ However, the current evidence base does not prove or disprove this theory, which limits the information available to patients prior to their surgery.

For both voice and swallowing outcomes, there is a similar need to design research with improved methodological quality, in particular, studies with a prospective design and consistent time points, and the use of outcome measures that consider reliability, validity, and responsiveness to change.

The number of studies that met the criteria for the systematic review is an indication that consideration of voice and swallowing outcomes in this patient group is clinically relevant. However, the lack of formalized outcome measures or consistent measurement of voice and swallowing is a major limitation in providing an evidence base to clinicians about these aspects of laryngotracheal stenosis and reconstructive surgery.

A prospective, standardized treatment protocol should include presurgical baseline assessment combined with postsurgical time points, with a clear rationale for outcome‐measure selection. Outcome measures should combine patient‐reported questionnaires such as the Voice Handicap Index‐10^46^ and Eating Assessment Tool‐10^47^; sensitive, instrumental assessments of dysphagia and dysphonia, such as Fiberoptic Endoscopic Evaluation of Swallowing or Modified Barium Swallow and acoustic analysis of voice; and clinician‐reported perceptual assessments such as the Grade‐Roughness‐Breathing‐Aesthenia‐Strain score,^48^ Consensus Auditory Perceptual Evaluation of Voice,^49^ or Functional Oral Intake Score.^50^ Future research needs to focus on developing this standardized treatment protocol for patients with laryngotracheal stenosis undergoing reconstructive surgery and including validated, reliable and responsive outcome measures. This will allow researchers to carry out studies that provide clearer answers to clinicians working in this area, and patients living with the condition and its treatment.

## CONCLUSION

This systematic review demonstrates that both voice and swallowing outcomes are thought to be of clinical relevance to the reconstructive treatment of laryngotracheal stenosis. Laryngotracheal stenosis is a rare, multifactorial disease, with a range of treatment options from the minimally to maximally invasive. Surgical treatment is not consistent from center to center,^51^ and the available research reviewing the efficacy of reconstructive approaches focuses on the primary outcome of improved airway/dyspnea. There is increasing acknowledgment in the literature that voice and swallowing are impacted by disease and treatment^10^, ^11^ however, this review shows a lack of consensus or rationale in selecting outcome measures for use in this population. Future research in this area should focus on well‐designed prospective studies, with an aim to create a core outcome metric for voice and swallowing to provide a “standardized collection of robust appropriate outcomes that could be measured and reported as a minimum”^52^ across all centers managing patients with laryngotracheal stenosis.
